# Abdominal manifestations of IgG4-related disease: a pictorial review

**DOI:** 10.1007/s13244-018-0618-1

**Published:** 2018-04-25

**Authors:** Christopher Siew Wai Tang, Nishanth Sivarasan, Nyree Griffin

**Affiliations:** Department of Radiology, Guy’s and St Thomas’ NHS Foundation Trust, Westminster Bridge Road, London, SE1 7EH UK

**Keywords:** Autoimmune pancreatitis, IgG4, Immunoglobulin G, Autoimmune diseases/diagnosis

## Abstract

**Abstract:**

In the last decade, autoimmune pancreatitis has become recognised as part of a wider spectrum of IgG4-related disease, typically associated with elevated serum IgG4 levels and demonstrating a response to corticosteroid therapy. Radiologically, there is imaging overlap with other benign and neoplastic conditions. This pictorial review discusses the intra-abdominal manifestations of this disease on cross-sectional imaging before and after steroid treatment and the main radiological features which help to distinguish it from other key differentials.

**Teaching Points:**

•* Autoimmune pancreatitis is part of a spectrum of IgG4-related disease.*

•* Diagnosis is based on raised serum IgG4, clinical, radiological and histopathological findings.*

•* Cross-sectional imaging can demonstrate the typical findings of abdominal IgG4-related disease.*

•* Cross-sectional imaging can be used to monitor response to corticosteroid treatment.*

## Introduction

Autoimmune pancreatitis (AIP) is a rare form of chronic pancreatitis secondary to an immune-mediated fibroinflammatory process, associated with elevated serum immunoglobulin 4 (IgG4) levels. AIP typically presents with abdominal pain and jaundice, and classically demonstrates a good response to corticosteroid therapy. In recent years, through better understanding of the pathophysiology of the condition, AIP has been reclassified within the wider spectrum of IgG4-related disease, with known associations including neurological, ocular, cardiovascular, respiratory and abdominal manifestations of disease.

The concept of AIP was first suggested in 1995 by Yoshida et al. [1], who described AIP as a chronic form of pancreatitis with an autoimmune aetiology. The link between AIP and raised serum IgG4 was then observed by Hamano et al. [2] in 2001, before Kamisawa et al. [3] proposed the concept of AIP as part of a disease spectrum of systemic IgG4 disease in 2003.

AIP is a rare disorder. Epidemiological data available from Japan estimates a prevalence of AIP of between 0.82–2.2 per 100,000 with a 2.9–3.7:1 male:female ratio, and typically affecting individuals older than 50 years of age.

Current understanding has led to the division of AIP into two subtypes. The key features are summarised in Table 1. Type 1 AIP is a manifestation of a systemic IgG4-related disease, as evidenced by the presence of histologically identical synchronous or metachronous lesions in other organs such as the salivary glands, bile ducts and kidneys [4, 5]. Type 2 AIP is not IgG4 mediated and is typically limited to the pancreas, although there is a known association with chronic inflammatory bowel disease [6]. Both subtypes respond well to corticosteroids, although there is a higher rate of recurrence in Type 1 patients, who are thought to benefit from long-term low-dose steroids [7].

**Table 1:** The key epidemiological, clinical and pathological differences between type 1 and type 2 autoimmune pancreatitis

	Type 1 AIP	Type 2 AIP
Gender	Male > female	Male = Female
Age	Older (6th decade)	Younger (4th decade)
Clinical Presentation	Painless obstructive jaundice	Painless obstructive jaundice, acute pancreatitis and abdominal pain
Histology	IgG4-rich periductal lymphoplasmocytic infiltrates	Granulocyte epithelial lesions (GEL)
Serum IgG4	Usually elevated	Normal
Extrapancreatic involvement	Salivary glands, biliary tree, kidneys, retroperitoneum	None
Treatment outcome	Excellent response to steroid, but recurrence is common	Excellent response to steroid, and recurrence is rare

Current diagnostic criteria for IgG4-related disease are based on a combination of three factors: (1) clinical examination (including clinical history, physical examination and imaging), (2) immunological examination (serum IgG4 >135 mg/dL or elevated IgG4/IgG ratio) and (3) histopathological examination (showing lymphoplasmocytic infiltration with storiform fibrosis and obliterative phlebitis, with infiltration by IgG4+ plasma cells) [8–10].

**Fig. 1:**
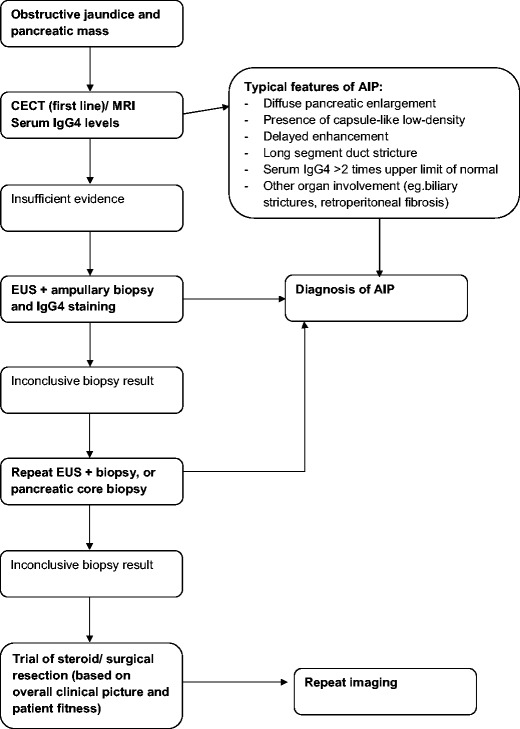
Flow chart showing suggested algorithm for distinguishing pancreatic cancer from AIP

Radiologically, IgG4-related disease can mimic other conditions, depending on which organ systems are involved. For example, pancreatic involvement can be mistaken for pancreatic adenocarcinoma, lymphoma, acute or chronic pancreatitis. An understanding of the pattern of presentation on cross-sectional imaging is essential in helping to make the correct diagnosis and direct appropriate management. A suggested algorithm for the workup and diagnosis of AIP is outlined in Fig. 1, based on our own local practice and published guidelines [11–13].

Between 2008 and 2016, 28 patients with the established diagnosis of AIP underwent cross-sectional imaging at our institution. From this database, this pictorial review describes the multi-modality imaging features of AIP and its extrapancreatic abdominal manifestations, both before and after treatment, with a focus on the key features that can help distinguish it from its main differential diagnoses.

## Imaging findings

### Pancreatic involvement

The morphological appearances of Type 1 and Type 2 AIP are radiologically indistinguishable. AIP may present with a diffuse form, a more focal form or a multifocal form. The key imaging features of AIP are summarised in Table 2, with particular reference made to findings that can distinguish AIP from pancreatic cancer.

**Table 2:** The main distinguishing features between AIP and pancreatic cancer on cross-sectional imaging

	AIP	Pancreatic cancer
CT	Peripancreatic hypoattenuating capsule (“halo”) present	Peripancreatic halo not present
	No upstream duct dilatation	Abrupt upstream duct dilatation often seen ± distal pancreatic atrophy
	Pancreatic duct wall enhancement sometimes present	No pancreatic duct wall enhancement
	Persistent enhancement in delayed phases	No delayed phase enhancement
	Homogenous enhancement pattern	Ring-like enhancement pattern
MRI	Low T1/T2 signal peripancreatic capsule	No peripancreatic capsule
	Duct narrowing occurs over a relatively long segment	Duct narrowing occurs over a shorter segment
	“Duct-penetrating sign” may be present	“Duct-penetrating sign” does not occur
	Restricted diffusion with low ADC values	Restricted diffusion, but ADC values are not as low as AIP
PET/CT	Heterogeneous and diffuse FDG uptake	Focal nodular FDG uptake
	Increased FDG uptake at extrapancreatic sites of disease	No extrapancreatic FDG uptake (unless metastatic to nodes or distant organs)

*AIP* autoimmune pancreatitis, *ADC* apparent diffusion coefficient, *CT* computed tomography, *FDG* fluorodeoxyglucose, *MRI* magnetic resonance imaging, *PET* positron emission tomography

#### CT imaging findings

Contrast-enhanced CT (CECT) is an important modality in the evaluation of AIP, and may clearly demonstrate classical features such as diffuse or focal pancreatic enlargement.

**Fig. 2:**
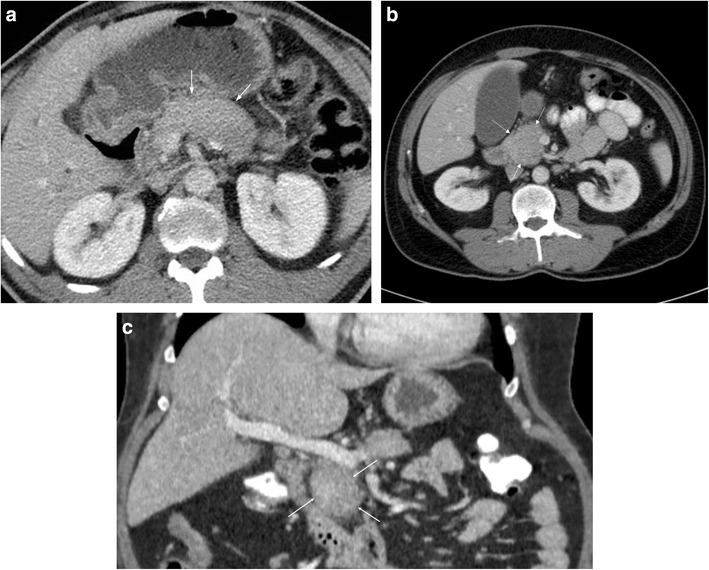
**a** CECT showing example of diffuse AIP with sausage shaped configuration to the pancreas and subtle peripancreatic halo of low attenuation (*arrows*). **b** Axial CECT in a different patient showing example of focal AIP affecting the head of the pancreas (*arrows*). **c** Coronal CECT in the same patient (Fig. 2**b**) demonstrating focal pancreatic head involvement (*arrows*)

The diffuse form is characterised by smooth, sausage-like enlargement of the pancreas with loss of the normal pancreatic lobulations (Fig. 2a). The involved pancreas tends to be hypoattenuating relative to normal parenchyma on CT, and there is usually preservation of peripancreatic fat planes, without vascular encasement; although there may be narrowing of peripancreatic veins. There is typically regional lymph node enlargement, which is a relatively non-specific feature. A more specific finding is the presence of a peripancreatic hypoattenuating capsule (Fig. 2a) (described as a “halo” by some sources), which is thought to represent a combination of fibrotic tissue, fluid and phlegmon. The presence of this peripancreatic capsule has been suggested to be a useful distinguishing feature between AIP and pancreatic cancer [14]. Notably, AIP is not usually associated with pancreatic pseudocysts, peripancreatic collections or retroperitoneal fluid, and the lack of these features may favour AIP over acute or chronic pancreatitis.

In its focal form, AIP may present with a well-defined focal mass-like lesion (Fig. 2b, c), causing irregular narrowing of the pancreatic duct, common bile duct (CBD) involvement and pancreatic tail retraction, in a manner that may mimic focal pancreatic cancer. A useful discriminator is the absence of upstream pancreatic duct dilatation in AIP, even in the presence of duct narrowing, and this feature may be appreciable on CT [15]. Furthermore, AIP is associated with main pancreatic duct wall enhancement, known as the “enhanced duct sign”, which is thought to occur due to periductal inflammatory changes and fibrosis [16, 17]. Although not common, this sign is thought to be specific for AIP, particularly in the focal form [18].

Post contrast, in early phase imaging there is classically decreased enhancement within affected regions relative to normal pancreatic parenchyma, with moderate and persistent enhancement during later delayed phases. It has been suggested that delayed phase images are useful for differentiating AIP and pancreatic cancer, with higher delayed phase attenuation values seen in the former [15, 19, 20]. AIP typically demonstrates a homogenous enhancement pattern, whereas pancreatic cancer may demonstrate ring-like enhancement, if enhancement occurs at all [18]. In addition, delayed enhancement of a peripancreatic capsule is highly suggestive of AIP [21].

#### MRI imaging findings

**Fig. 3:**
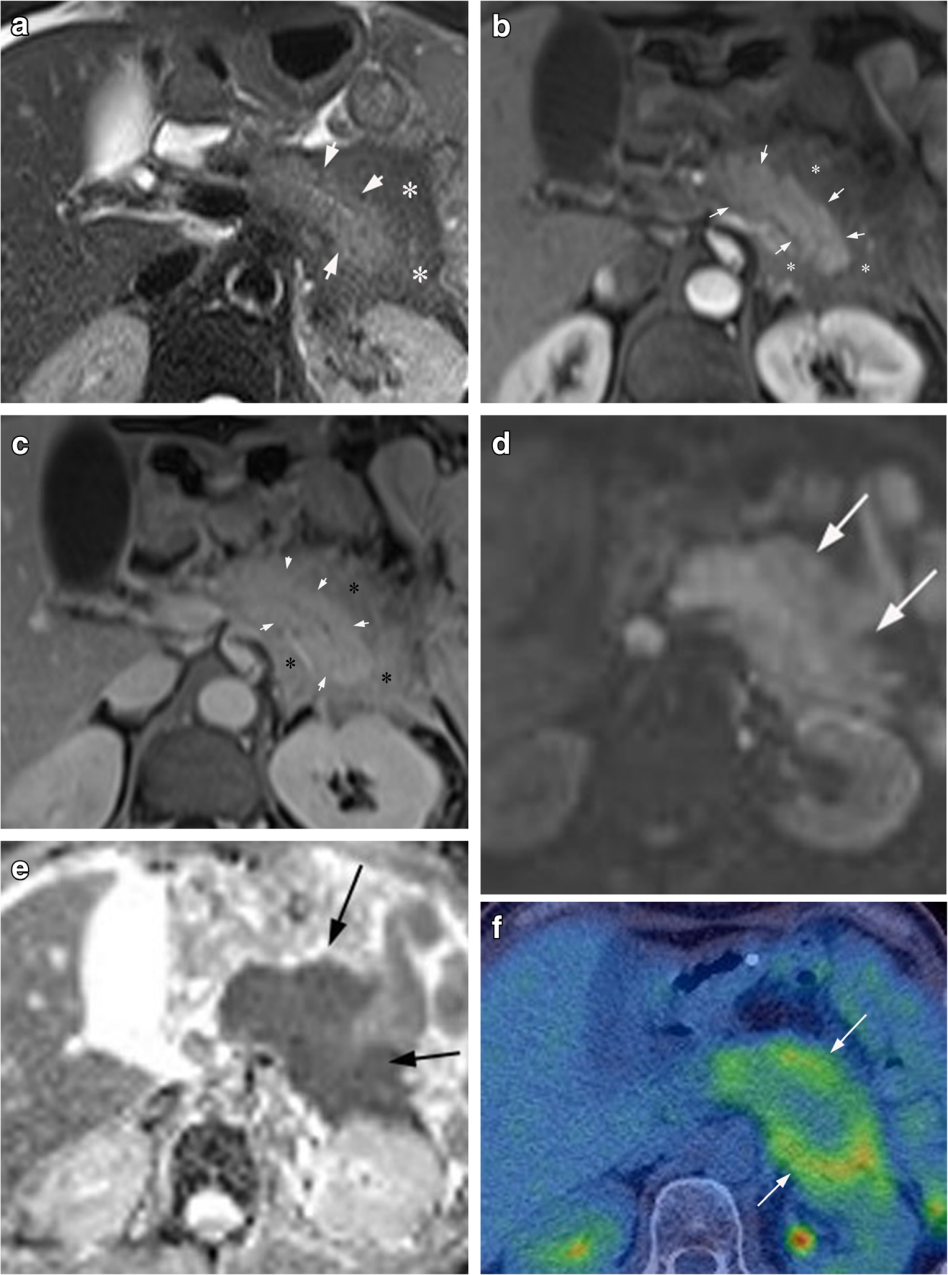
A patient with a history of mucosa-associated lymphoid tissue (MALT) lymphoma, presenting with raised serum IgG4 and diagnosis of AIP on endoscopic ultrasound (EUS)-guided biopsy. **a** Axial T2 fat saturated (FS) turbospin echo image shows intermediate to high T2 signal within the pancreas (*arrows*), but extensive low T2 signal peripancreatic tissue (*). There is diffuse narrowing of the distal pancreatic duct. **b** Axial T1FS post-contrast arterial phase initially shows reduced enhancement of the peripancreatic tissue (*) compared to the pancreas (*arrows*). **c** Axial T1FS post-contrast equilibrium phase showing delayed enhancement of the peripancreatic tissue. **d** The pancreas and extrapancreatic soft tissue shows marked restricted diffusion with high signal on diffusion-weighted imaging—B800 (*arrows*), a typical finding in AIP. **e** The tissue demonstrates corresponding low signal on the ADC map (*arrows*). **f** Only the extrapancreatic soft tissue shows high-grade FDG uptake (*arrows*), a finding which was felt to be atypical for lymphoma

**Fig. 4:**
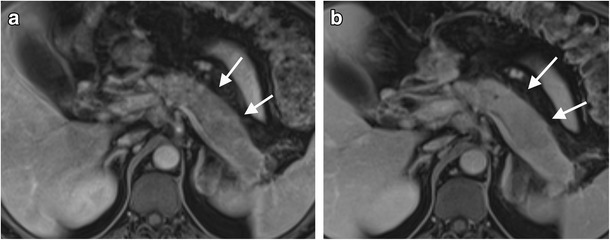
Axial T1FS post-contrast imaging demonstrating late enhancement in diffuse AIP. **a** The pancreas is relatively hypointense in the arterial phase (*arrows*). **b** The pancreas shows more avid enhancement in the portovenous phase (*arrows*)

MRI imaging of AIP (Figs. 3 and 4) demonstrates the same gross morphological features as CT; diffuse or focal pancreatic enlargement with loss of normal pancreatic lobulations. Areas involved by AIP demonstrate hypointense T1 and hyperintense T2 signal, whereas the low attenuation peripancreatic capsule demonstrates hypointense signal on both T1 and T2 sequences [22, 23]. Dynamic contrast-enhanced MRI demonstrates a similar pattern of enhancement as seen on CT (non-enhancement on the early phase, with enhancement seen on later phases) (Figs. 3b, c and 4a, b). Similarly, the peripancreatic capsule demonstrates delayed enhancement [24].

The use of T2-weighted sequences (with or without fat suppression) on MRI can better delineate the pancreatic duct narrowing compared to CT, due to better contrast resolution (Fig. 3a). As discussed previously, AIP may demonstrate irregular duct narrowing without associated upstream dilatation [25, 26]. In addition, the irregular duct narrowing of AIP is typically over a longer segment compared to duct narrowing seen in pancreatic cancer [27]. These pancreatic duct appearances have been corroborated with endoscopic retrograde cholangiopancreatography (ERCP) findings, with one multicentre study suggesting that four key features on ERCP are: (1) long (more than one-third the length of the pancreatic duct) stricture; (2) lack of upstream dilatation (<5 mm); (3) multiple strictures; (4) side branches arising from a strictured segment [28]. Secretin-enhanced MRCP improves duct distension and has been described as a potentially useful problem-solving tool in the diagnosis of AIP.

Some studies have suggested that the presence of a non-obstructed main pancreatic duct passing through the ‘mass’ may be useful as a discriminator between an inflammatory pancreatic mass and pancreatic cancer, with the ‘duct penetrating sign’ more commonly seen in the former [20, 29].

Diffusion-weighted imaging (DWI) and the corresponding apparent diffusion coefficient (ADC) can be useful in evaluating AIP (Fig. 3d, e). Both AIP and pancreatic cancer demonstrate increased signal on high *b*-value sequences, with corresponding low ADC values. One study reported that AIP demonstrates high *b*-value DWI signal in a more linear morphology compared to pancreatic cancer [30]. In addition, multiple studies have observed that AIP typically demonstrates lower ADC values compared to pancreatic cancer, although both have low values relative to normal pancreatic parenchyma [20, 25, 31].

#### PET/CT imaging findings

**Fig. 5:**
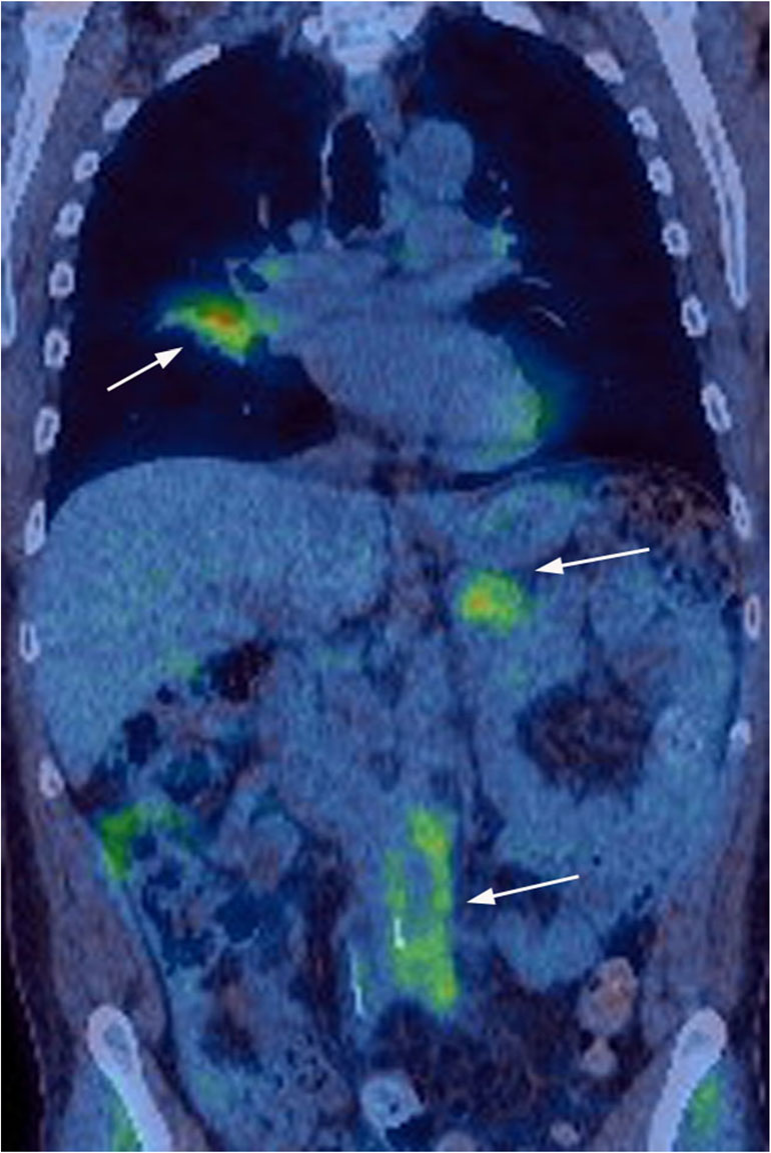
Coronal FDG-PET/CT study showing multifocal IgG4 disease with involvement of the right lung, tail of the pancreas and retroperitoneum (*arrows*)

FDG PET/CT imaging has been shown to have utility for the diagnosis of AIP (Figs. 3f and 5). Multifocal increased FDG uptake has been shown to be sensitive, although not specific, for AIP [32, 33]. While FDG avidity may also be seen in pancreatic cancers, typical chronic pancreatitis does not usually demonstrate FDG uptake. Furthermore, it has been reported that the pattern of FDG avidity may help discriminate between AIP and pancreatic cancer, with a diffuse, elongated and heterogenous pattern of uptake thought to favour AIP (in contrast to a nodular focal pattern favouring pancreatic cancer) [32, 33]. A more recent study has suggested that the measurement of SUV ratios between the pancreas and liver may also be helpful in distinguishing AIP and cancer, with pancreatic lesions in AIP demonstrating a lower maximum standard uptake value (SUV) ratio compared to pancreatic cancer [34]. In addition, FDG PET/CT is excellent at demonstrating extrapancreatic sites of disease (Fig. 5), which, when present, are strongly suggestive of a diagnosis of AIP over pancreatic cancer.

#### Response to treatment imaging findings

**Fig. 6:**
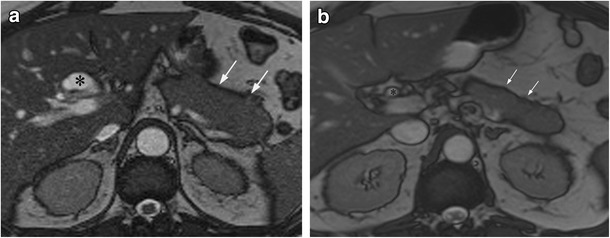
**a** Axial T2 balanced gradient echo image shows diffuse enlargement of the body and tail of the pancreas (*arrows*) with proximal biliary dilatation due to a distal common bile duct (CBD) stricture (*). **b** Following corticosteroid treatment, the pancreas is less bulky (*arrows*) and the biliary dilatation is no longer prominent (*) due to resolution of the previous biliary stricture (not shown)

AIP is characterised by an excellent response to treatment with corticosteroid therapy, both clinically and radiologically, with radiological improvement usually seen within 2 weeks (Figs. 6a, b). Multiple studies have demonstrated resolution of imaging abnormalities after treatment on CT and MRI. Key features of treatment response include reduction in the size of the pancreatic parenchyma, normalisation of pancreatic enhancement characteristics and normalisation of the pancreatic duct diameter [21, 23, 35]. It has been reported that treatment response can be predicted by the stage of disease. Features of early phase inflammation, such as diffuse swelling and a peripancreatic capsule, are predictors of good response to corticosteroid therapy, whereas features of the later fibrotic phase of disease, such as focal mass-like swelling and ductal strictures, are predictors of a poorer response to treatment [35]. FDG PET/CT can also be used to monitor treatment response, with multiple studies demonstrating a reduction in FDG uptake and maximum SUV after appropriate corticosteroid treatment [33, 36]. Steroid-responsiveness is unique to autoimmune pancreatitis and repeat imaging of AIP after corticosteroid treatment is therefore invaluable in establishing the diagnosis. The 2011 international consensus guidelines recommend repeat imaging after a 2-week trial of corticosteroid treatment in the context of a new diagnosis of AIP [37].

### Biliary involvement

**Fig. 7:**
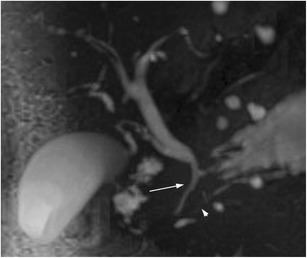
Example of IgG4 biliary stricture: MRCP shows a long smooth stricture involving the intrapancreatic portion of the CBD (*arrow*). The proximal pancreatic duct is also attenuated (*arrowhead*)

**Fig. 8:**
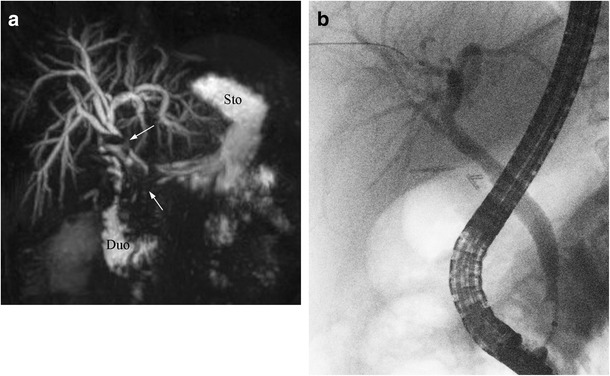
Example of IgG4-related biliary stricture in a different patient. **a** Strictures are seen in the common hepatic duct and distal CBD (*arrows*). *Sto* stomach, *Duo* duodenum. **b** ERCP performed 6 months later on the same patient shows resolution of the extrahepatic strictures after treatment with corticosteroids

Biliary tree involvement is seen in the majority (up to 90%) of patients with AIP, and presents in the form of IgG4 cholangitis (Figs. 7 and 8). Evaluation of biliary involvement in AIP can be performed via CT, magnetic resonance cholangiopancreatography (MRCP) or ERCP, and characteristic features include stricturing and narrowing of the bile ducts, bile duct fibrosis and bile duct wall thickening. The key differential diagnosis is primary sclerosing cholangitis, which usually affects a younger patient demographic. Biliary strictures in IgG4 disease are typically long and smooth in morphology, with associated upstream dilatation, and patients may consequently develop obstructive jaundice. This is in contrast to the appearances of primary sclerosing cholangitis, which demonstrates multifocal short, band-like strictures that lead to a “beaded” appearance [38].

Bile duct wall thickening in IgG4 cholangitis is readily appreciable on cross-sectional imaging, and demonstrates enhancement on post-contrast studies. Although both the intrahepatic and extrahepatic bile ducts can be affected, the most common site of involvement is the intrapancreatic segment of the CBD [39]. The gallbladder wall may also be involved, demonstrating wall thickening and enhancement [40, 41]. As with the pancreatic findings of AIP, IgG4 cholangitis can demonstrate a good response to corticosteroid treatment, and this is reflected radiologically by the resolution of biliary strictures and bile duct wall thickening (Figs. 8a, b) [38, 42].

### Renal involvement

**Fig. 9:**
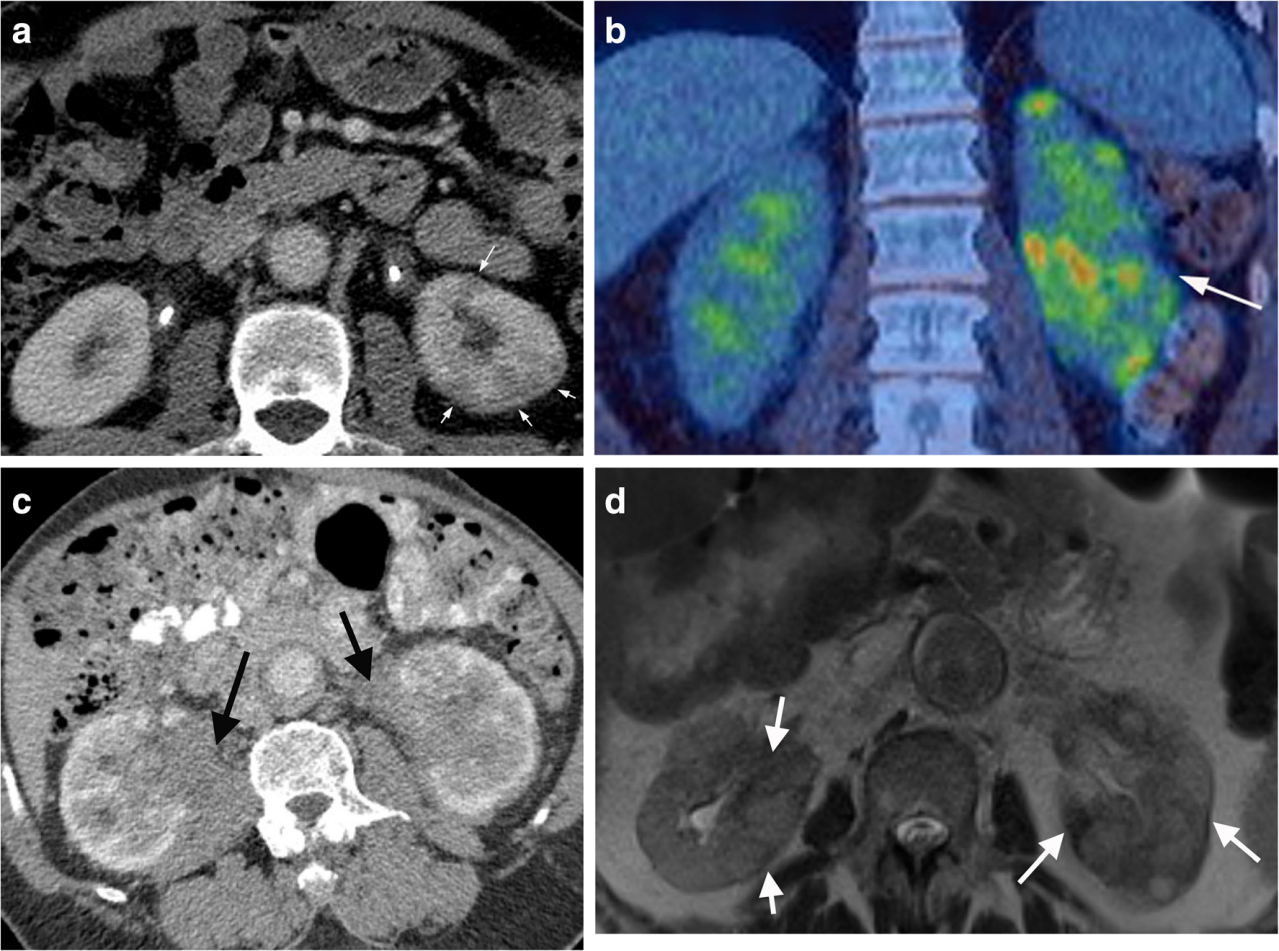
Example of IgG4 disease affecting the left kidney. **a** Axial CECT shows typical wedge-shaped low-density renal cortical lesions in the left kidney (*arrows*). Both kidneys are stented as there was also IgG4 related retroperitoneal fibrosis. **b** Coronal FDG PET/CT in same patient as in **a** showing multifocal high-grade uptake in the left kidney (*arrow*). **c** Example of bilateral renal pelvic involvement in a different patient (*arrows*) on CECT. **d** Axial T2 turbospin echo image in different patient shows renal lesions (*arrows*) are of mixed high and low T2 signal (typically reported in the literature as low T2 signal)

**Fig. 10:**
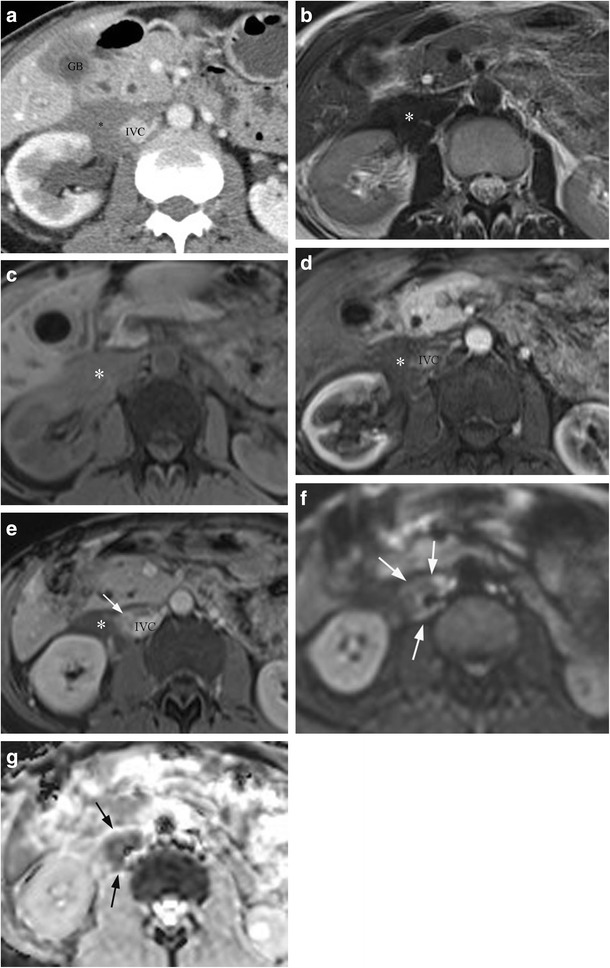
Example of extrarenal IgG4 disease. **a** CECT showing a rind of abnormal soft tissue (*) anterior to the right kidney and encasing the IVC. **b)** Axial T2 turbo spin echo image of the same patient as in Fig. 9a shows perirenal soft tissue is of low T2 signal (*). **c** Axial T1FS pre-contrast shows perirenal soft tissue of intermediate T1 signal (*). **d** Axial T1FS post-contrast arterial phase shows minimal enhancement of the perirenal soft tissue (*). **e** Axial T1FS post-contrast equilibrium phase shows delayed enhancement related to a cuff of soft tissue around the IVC (*arrow*) with most of the perirenal soft tissue remaining low T1 signal (*). **f** The enhancing tissue around the IVC shows restricted diffusion with high signal on DWI—B800 (*arrows*). **g** There is corresponding low signal on the ADC map (*arrows*)

Renal involvement is frequently seen with AIP, and renal parenchymal involvement is noted around 30% of patients with AIP [43]. There are several patterns of renal involvement which have been described; these include generalised renal enlargement, wedge-shaped or rounded parenchymal lesions (usually located in the renal cortex) (Figs. 9a, b), renal pelvic lesions (Fig. 9c, d), and the development of a perirenal soft tissue rind (Fig. 10).

Renal parenchymal lesions are the most common manifestation of renal IgG4 disease. On CT imaging, renal parenchymal lesions are typically isodense to renal cortex on pre-contrast imaging, but hypoattenuating on corticomedullary phase imaging (Fig. 9a), before developing gradual progressive enhancement on more delayed phases [44]. Renal pelvic lesions are far less common compared to renal parenchymal lesions, and are characterised by thickening and enhancement of the renal pelvis (Fig. 9c). The development of a perirenal soft tissue rind is a relatively rare manifestation of AIP (Fig. 10a) with delayed enhancement post-contrast.

On MRI, these lesions usually exhibit hypointense T2 and isointense T1 signal (Fig. 10b, c), with enhancement characteristics similar to those seen on CT (Fig. 10d, e). In addition, these lesions demonstrate increased signal on high *b*-value DWI sequences, with matching low ADC values (Fig. 10f, g), and it has been reported that DWI sequences may be superior to conventional MRI sequences in terms of early detection of subclinical parenchymal lesions [45].

Important differentials to consider when evaluating for renal IgG4 disease include metastatic disease, renal lymphoma and pyelonephritis. In all these cases, the presence of IgG4 disease in other organ systems should favour a diagnosis of renal IgG4 disease. Clinical correlation is also crucial; for example, metastases should be considered in the context of a known primary malignancy, and pyelonephritis should be considered in the context of a septic patient. Renal lymphoma may closely mimic parenchymal IgG4 disease in terms of MRI characteristics, but is often associated with significant retroperitoneal lymphadenopathy, in contrast to renal IgG4 disease. In addition, lymphoma is not associated with elevated serum IgG4 [45].

As with pancreatic and biliary IgG4 disease, renal IgG4 disease exhibits a good response to corticosteroid treatment, with improvement in imaging findings and biochemical renal function, although some patients may relapse whilst on treatment. Post-treatment appearances of successfully treated renal IgG4 disease include resolution of previously seen lesions, as well as cortical scarring in the corresponding locations [43].

### Retroperitoneal and other abdominal involvement

**Fig. 11:**
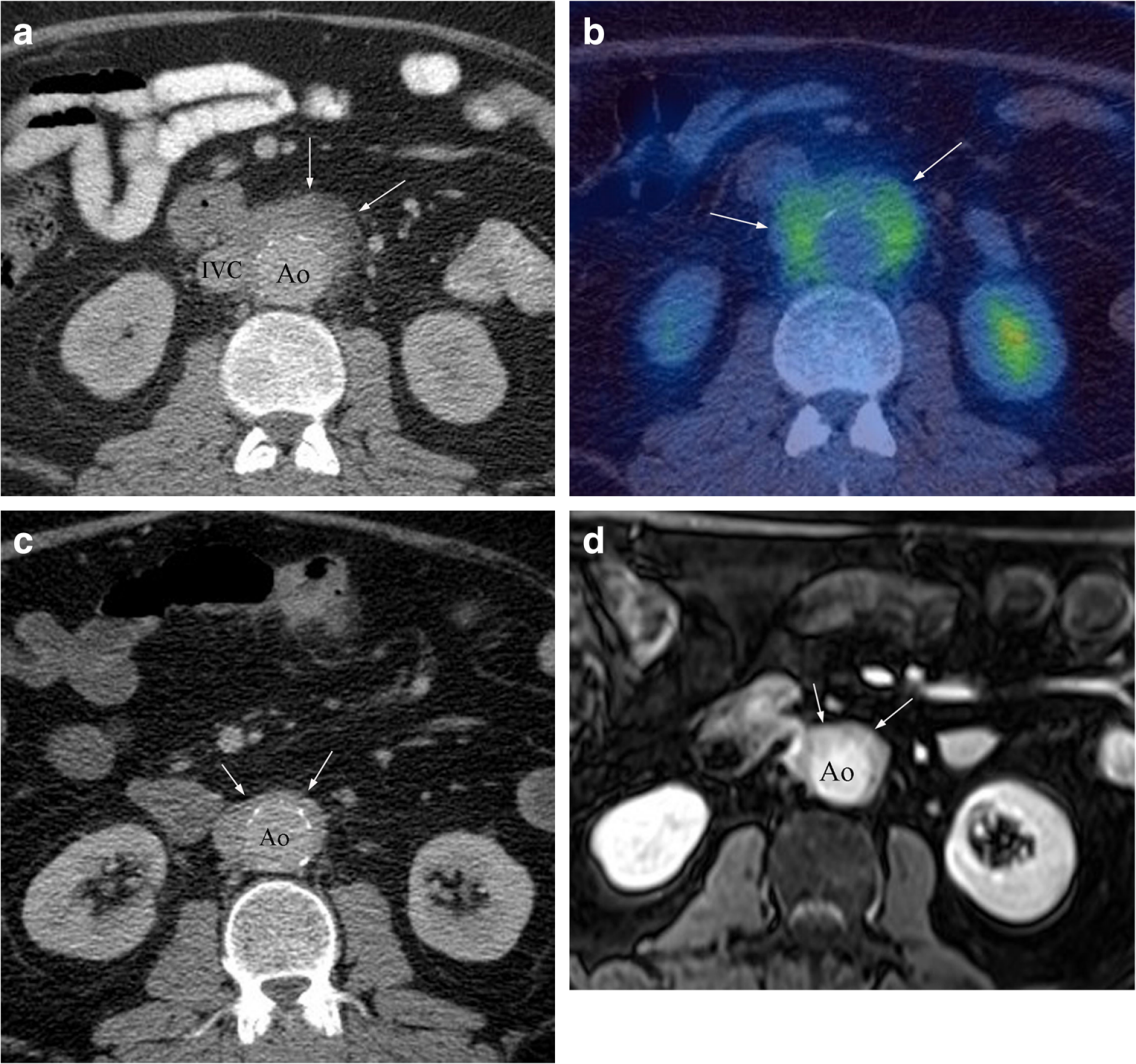
Example of IgG4 retroperitoneal fibrosis. **a** Axial CECT showing enhancing soft tissue partly encasing the IVC and aorta (*arrows*). **b** Axial FDG PET/CT showing increased FDG uptake in the abnormal retroperitoneal soft tissue (*arrows*). **c** Axial CECT in the same patient, with reduced periaortic soft tissue, following treatment with corticosteroids. **d** Corresponding T1FS post-contrast MRI showing residual enhancing soft tissue around the aorta following steroid treatment (*arrows*)

AIP-associated IgG4 disease is known to involve the retroperitoneum, principally in the form of retroperitoneal fibrosis, and less commonly in the form of periaortitis. An estimated 10% of patients with AIP develop retroperitoneal fibrosis, and this has similar imaging findings compared to retroperitoneal fibrosis secondary to other causes [22]. It manifests as a retroperitoneal mass which encases the aorta (Fig. 11) and may cause hydronephrosis secondary to the extrinsic compression of the ureters. On CT, the area of fibrosis appears as a soft-tissue mass with variable enhancement (Fig. 11a) [46]. MRI appearances include low/intermediate T1 signal, variable T2 signal and variable contrast enhancement, depending on the degree of inflammatory activity and fibrous tissue [39] On FDG PET/CT imaging, the affected areas exhibit avid FDG uptake (Fig. 11b). As with previously described IgG4 disease, there is typically complete or near complete resolution of imaging findings after treatment with corticosteroids (Fig. 11c, d).

Vascular involvement in IgG4 disease may include periaortitis. This is characterised by vessel wall thickening and enhancement, and if untreated may progress to dissection and aneurysm formation. On CT, the affected areas are apparent as wall thickening and late phase enhancement, and on FDG PET/CT it has been reported that affected areas demonstrate increased FDG uptake [47–49].

AIP has been reported in the literature to involve other abdominal organs, including the mesentery, abdominal lymph nodes, prostate, stomach and liver [39, 50, 51]. Involvement of these systems is very rare, and imaging features include soft-tissue masses that demonstrate PET avidity. Ultimately, diagnosis in these rare cases is reliant on biopsy and response to steroid treatment.

## Conclusions

AIP is an uncommon but important condition and is now recognised as a manifestation of systemic IgG4-related disease. Cross-sectional imaging (including the use of PET/CT), plays an essential role in the diagnosis of AIP, helping to establish the extent of involvement, and in the evaluation of treatment response. Common sites of involvement within the abdomen include the pancreas, biliary tract, kidneys and retroperitoneum. Depending on the site of involvement, imaging features may include focal or diffuse enlargement of the pancreas, smooth distal biliary strictures, focal renal involvement and/or abnormal periaortic or perirenal soft tissue. Typically, the sites affected show late enhancement, restricted diffusion on MRI and uptake on PET/CT, with resolution (or near resolution) of findings following corticosteroid therapy. It is hoped that this pictorial review has improved the understanding of the reader in recognising this important disease entity within the abdomen.

## Abbreviations

AIP: Autoimmune pancreatitis
CBD: Common bile duct
ERCP: Endoscopic retrograde cholangiopancreatography
IgG4: Immunoglobulin 4
MRCP: Magnetic resonance cholangiopancreatography
T1FS: T1 fat-saturated
